# Need for Critical Rethinking in Clinical Approaches to Neuroangiostrongyliasis

**DOI:** 10.4269/ajtmh.19-0546a

**Published:** 2019-10

**Authors:** Paul Prociv

**Affiliations:** Department Microbiology and Parasitology; University of Queensland; St. Lucia, Australia; E-mail: pmprociv@bigpond.com

Dear Sir,

The comprehensive and timely review by Johnson’s group^1^ provides useful information relating to the seasonal and geographic distribution of human neuroangiostrongyliasis (NA) in Hawaii. Given Hawaii’s prominence in the annals of research into *Angiostrongylus cantonensis*, it is particularly disturbing to witness the emergence there of such a potentially debilitating infection, now growing into a serious public health problem. However, I feel the review could have benefited clinicians further by analyzing the pathogenesis, diagnosis, and clinical management of NA in more depth.

First, the assumption that developing larvae of *A. cantonensis* all die in the human CNS overlooks growing evidence that many of these larvae actually develop fully and eventually leave the CNS to settle within pulmonary arteries, with significant pathological consequences.^2^ During their CNS sojourn, the larvae grow rapidly in bulk, by more than three orders of magnitude. As pathology presumably arises mainly from inflammatory responses to arrested and dead larvae, the timing of anthelmintic therapy may be critical.

Second, the statement “the use of anthelmintic drugs has been controversial with unclear benefits” reflects a prevailing view that must now be revisited, in light of a recent analysis of treatment timing.^2^ When used, these drugs tend to be given regardless of the stage of infection, potentially explaining their erratic efficacy. Beyond 3 weeks after exposure, when surviving larvae are large and likely to leave the CNS, anthelmintics might be contraindicated, serving to exacerbate local pathology. However, in earlier stages, with minute larvae, their use is more likely to be beneficial.^2,3^

Third, the diagnostic cutoff levels given for CSF cell findings could have been more specific. Whereas > 6 leukocytes per mm^3^ is widely accepted for diagnosing inflammation, what is critical is that, in normal CSF, these should be only lymphocytes; even finding one eosinophil is abnormal. Eosinophil counts, in both blood and CSF, should be presented as absolute numbers. To propose diagnostic eosinophil numbers as a percentage of total white cells, in either peripheral blood or CSF, may be misleading, as the factors controlling proliferation and mobilization of different granulocytes (neutrophil, eosinophil, and basophil) are largely independent.

Fourth, all available specific and sensitive diagnostic tests for NA, including blood and CSF serology (for antibodies against *A. cantonensis*), and even PCR (for parasite DNA), can be slow to convert, becoming positive sometimes as late as 3 weeks after the onset of infection. A consideration of parasite dynamics in the CNS offers logical explanations for why *A. cantonensis* DNA might not appear in the CSF earlier. Although it is understandable for clinicians to want a positive confirmation before commencing specific treatment, in the case of NA, delay may entail dire consequences for the patient, given larval growth rates: each passing day evidences compounding CNS damage and declining benefits from anthelmintics. It is perfectly reasonable, for diagnostic confirmation, to advise that “if a case has a compelling clinical and epidemiologic history strongly suggesting angiostrongyliasis but has a negative RTi-PCR result, we recommend the RTi-PCR test be repeated on a subsequent CSF specimen obtained at least a week after the initial specimen,” but it also must be stressed that if the infection is in its early stages, that is, within the 3-week “window of opportunity,” then anthelmintics should be commenced even before test results are available. Otherwise, the clinical outcome might be suboptimal.

Should eosinophilic meningoencephalitis be the provisional diagnosis, with early *A. cantonensis* infection not confidently excluded, then the working policy should be “shoot first, ask questions later.” A case could be made for treating all neurological presentations in which early NA fits among the differential diagnoses, in an appropriate epidemiological setting, urgently with anthelmintics.^2,3^ Albendazole is expensive in the United States, but it can be given for only a brief course in NA, perhaps as short as 5 days.^3^

Fifth, and understandably, given that most published case reports also downplay this aspect of the infection, the review overlooks the long-term sequelae of NA. However, personal feedback from clinicians in Hawaii and Australia suggests that this can be a significant problem, possibly affecting most clinical cases. For example, a patient whom I had described previously^2^ died of neurological and pulmonary complications, more than 8 years after becoming infected. In my experience, even patients with mild disease often complain for months or years afterward of localized motor and sensory abnormalities, suggesting permanent nerve damage. Clinicians in Hawaii are well placed to investigate this in the long term.

Finally, given that *A. cantonensis* does frequently develop to maturity in the human lungs, it would be fascinating, if only from a biological perspective, to determine if it can reproduce there.^2^ Again, researchers in Hawaii would seem ideally placed to observe this development, by following up all cases of human infection for the fecal passage of first-stage larvae beyond 2–3 months after their initial exposure to infection.

## References

[1] Johnston DI Dixon MC Elm JE Jr. Calimlim PS Sciulli RH Park SY, 2019 Review of cases of angiostrongyliasis in Hawaii, 2007–2017. Am J Trop Med Hyg 101: 608–616. 31287041 10.4269/ajtmh.19-0280 PMC6726938

[2] Prociv P Turner M, 2018 Neuroangiostrongyliasis: the “subarachnoid phase” and its implications for anthelminthic therapy. Am J Trop Med Hyg 98: 353–359. 29210355 10.4269/ajtmh.17-0206 PMC5929180

[3] Berkhout A Prociv P Anthony H Anthony LT Nourse C, 2019 Two cases of neuroangiostrongyliasis: a rare disease because rarely considered or rarely diagnosed? J Paediatr Child Health [Epub ahead of print 22 April 2019]. 10.1111/jpc.14461 30945367

